# Endoscopic diagnosis of *Fasciolopsis buski*: Revisited (with video)

**DOI:** 10.1002/jgh3.12187

**Published:** 2019-04-22

**Authors:** Ashish K Jha, Sharad K Jha

**Affiliations:** ^1^ Department of Gastroenterology Indira Gandhi Institute of Medical Sciences Patna India; ^2^ Department of Gastroenterology, Women and Gastro Center Darbhanga India

**Keywords:** *Fasciolopsis buski*, fascioliasis, endoscopy, mass chemotherapy

## Abstract

*Fasciolopsis buski* is the largest fluke parasitizing the human small intestine. *F. buski* infections are not uncommon in Southeast Asia. The risk factors of *F. buski* infection mainly include eating of raw aquatic crops and infected snails. Most infections are asymptomatic. Heavy infection can be fatal as the flukes cause extensive intestinal inflammation, intestinal perforation, small bowel stricture, ulceration, hemorrhage, and abscess formation. Endoscopic diagnosis of this parasite has been described in a few case reports. Here, we describe and illustrate the endoscopic removal of *F. buski* from the stomach and duodenum.

## Graphical abstract

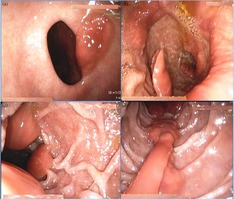

Endoscopic diagnoses of human *Fasciolopsis buski* mostly come as a surprise, especially in the absence of intense peripheral eosinophilia. Although *F. buski* infections are not uncommon in Southeast Asia, endoscopic diagnosis of parasite has been described in a few case reports. Here, we describe and illustrate the endoscopic removal of *F. buski* from the stomach and duodenum.

## Introduction

*Fasciolopsis buski* is the largest fluke parasitizing the human small intestine.^1^ Although *F. buski* infections are not uncommon in Southeast Asia, endoscopic diagnosis of this parasite has been described in a few case reports.^2^, ^3^, ^4^ Here, we describe and illustrate the endoscopic removal of *F. buski* from the stomach and duodenum.

## Case report

**Figure 1:**
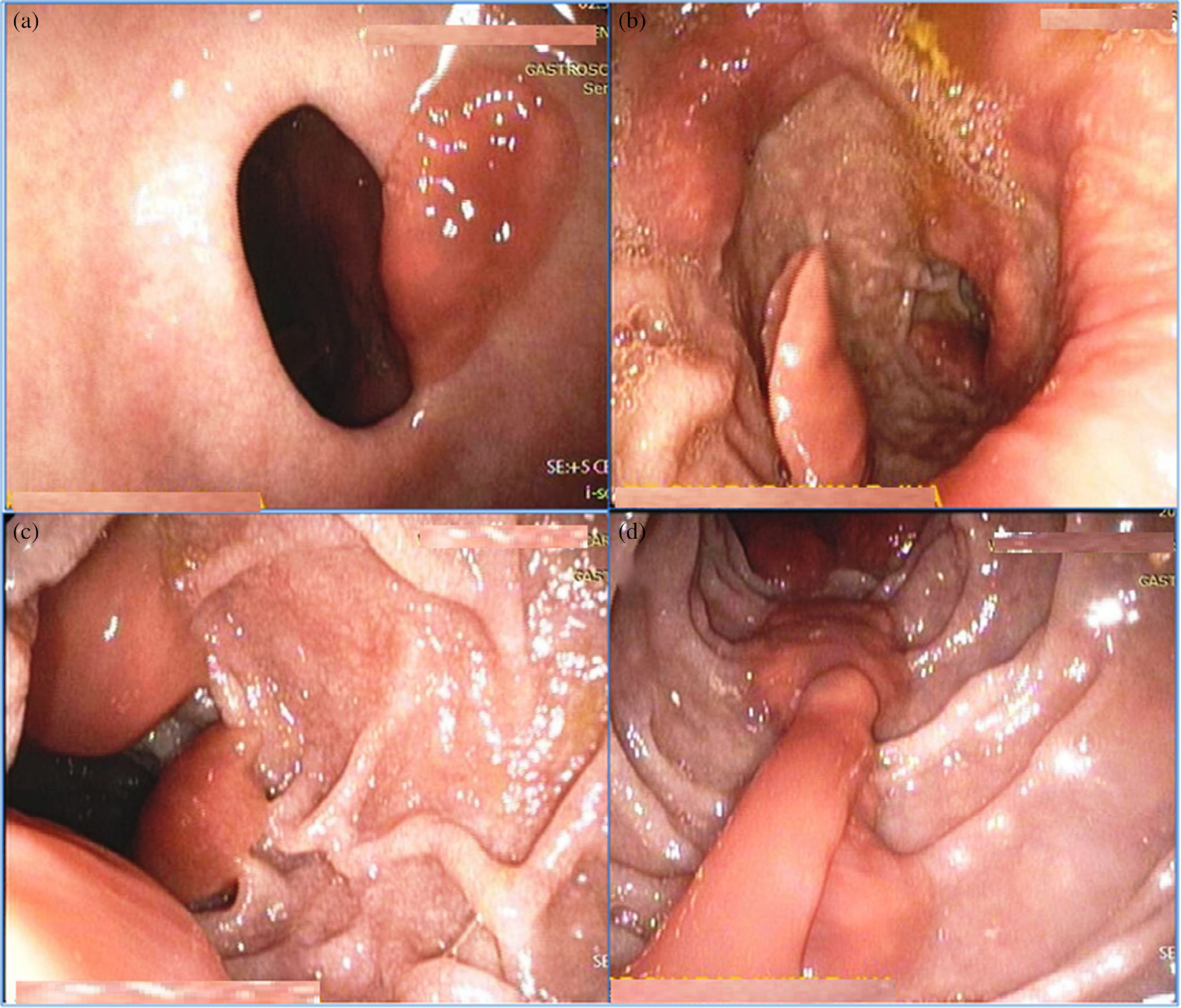
Endoscopic view of multiple *Fasciolopsis buski* stuck in the pyloric antrum (a) and the first (b) and second part of the duodenum (c, d).

A 40‐year‐old Indian female presented with a 2‐week history of epigastric pain, vomiting, and passage of flesh‐like material in the stool. She provided a history of consumption of raw *singhada* (water caltrop). She used to wash the kitchen utensils and raw vegetables in pond water. Blood examination showed hemoglobin of 10.5 g/dL and normal total eosinophil count. Stool examination was normal. Esophagogastroduodenoscopy showed multiple live flatworms attached to the pyloric and duodenal mucosa (Fig. 1). Worms were variable in size and reddish‐brown in color. Endoscopic extractions of some of the worms were performed with the help of biopsy forceps. They were fleshy, reddish brown, dorsoventrally flattened, and leaf‐like, measuring 4.0 cm in length, 2.5 cm in breadth, and 2.5 mm in thickness with no prominent or obvious cephalic cone, resembling *F. buski* (Video Clip S1, Supporting information). Microscopic examination of an adult worm confirmed *F. buski*. The patient was treated with praziquantel 75 mg/kg in three divided doses for 1 day. The patient was asymptomatic and doing well on follow‐up.

## Discussion

Among the zoonotic parasitic diseases, human fascioliasis is classified as a plant‐/food‐borne trematode infection. *F. buski* is endemic to Bangladesh, China, Taiwan, India, Indonesia, Malaysia, Thailand, the Lao People's Democratic Republic, and Vietnam.^1^ Fasciolopsis is an example of a parasitic disease that is emerging (or re‐emerging) in many countries as a consequence of changes in both environmental and human factors.^1^, ^5^, ^6^ *F. buski* remains a public health problem in endemic regions.^7^ Because of population migration and international food trade, human fascioliasis is an increasingly recognized entity in nonendemic zones.^8^

The lifecycle of *F. buski* requires mammalian final hosts (human or pigs), snails (intermediate host), and aquatic plants such as water chestnut, water caltrop, lotus, bamboo, and other edible water plants. The monoecious adult worms live in the proximal small gut of humans and pigs. Humans are infected through ingestion of encysted infective metacercariae, which are found adhering to the surface of edible water plants. After excystation, juvenile worms migrate to the proximal small intestine, grow to maturity, and produce eggs that pass out of the host in excreta. If stool of infected host enters fresh water, the eggs hatch, releasing immature larvae (miracidia), which enter snails to begin the cycle again. Within the infected snail, the miracidia then transform to free‐swimming cercariae. The cercariae adhere to and encyst, as metacercariae, on vegetation and are infective to the definitive host.

*F. buski* infections are not uncommon in northern Bihar (India).^9^, ^10^ Water caltrop/singhada (*Trapa natans*), lotus, and makhana/fox nut (*Euryale ferox*) are the aquatic crops cultivated in northern Bihar. The risk factors for *F. buski* infection may include practice of open defecation in the open field, river bank, and alongside ponds; eating of raw aquatic crops and infected snails; and washing of the kitchen utensils and raw vegetables in pond water.

Most infections are asymptomatic. Symptoms include abdominal pain, diarrhea, fever, gastrointestinal bleeding, ascites, anasarca, and intestinal obstruction. Heavy infection can be fatal as the flukes cause extensive intestinal inflammation, intestinal perforation, small bowel stricture, ulceration, hemorrhage, and abscess formation.^1^, ^2^ It is usually diagnosed by identification of the characteristic bile‐stained egg with operculum at one end (in normal saline mount) in the stool or vomitus. The morphology of eggs is similar to those of *Fasciola hepatica*. Definite diagnosis requires the examination of adult worms. *F. buski* infestation is treated with praziquantel, 75 mg/kg/day orally, in three divided doses for 1 day.

In conclusion, live *F. buski* may be found during endoscopy, and they can be extracted with the help of a foreign body retriever or biopsy forceps. The training of public health professionals, health education in a high‐risk population, and mass chemotherapy in endemic regions are the different measures used for the prevention and control of the disease.

## Supporting information


**Video Clip S1.** Showing live, motile *Fasciolopsis buski* extracted from the duodenum.
